# Synthesis and Cytotoxicity of Chalcones and 5-Deoxyflavonoids

**DOI:** 10.1155/2013/649485

**Published:** 2013-06-06

**Authors:** Jing Zhang, Xin-Ling Fu, Nan Yang, Qiu-An Wang

**Affiliations:** ^1^College of Chemistry and Chemical Engineering, Hunan University, Changsha 410082, China; ^2^Department of Basic Medical, Changsha Medical University, Changsha 410082, China

## Abstract

Chalcones **1~8** and 5-deoxyflavonoids **9~22** were synthesized in good yields by aldol condensation, Algar-Flynn-Oyamada reaction, glycosidation, and deacetylation reaction, respectively, starting from 2-acetyl phenols substituted by methoxy or methoxymethoxy group and appropriately benzaldehydes substituted by methoxy, methoxymethoxy group, or chlorine. Among them, **13** and **17~22** are new compounds. The cytotoxicity bioassays of these chalcones and 5-deoxyflavonoids were screened using the sulforhodamine B (SRB) protein staining method, and the results showed that compounds **2, 4, 5, 6, 10, 15**, and **19** exhibited moderate cytotoxicity against the cancer cell line of MDA-MB-231, U251, BGC-823, and B16 in comparison with control drugs (HCPT, Vincristine, and Taxol).

## 1. Introduction

Chalcones are a class of natural compounds that widely exist in a variety of plant species. Chemically, they consist of open-chain flavonoids in which the two aromatic rings are joined by a three carbon *α*, *β*-unsaturated carbonyl system. The flexible structure of chalcones makes them have a large number of biological activities including antitumor [1], antifungal [2]_,_ antiprotozoal [3], antimitotic [4], and antivirus [5] properties. The 5-deoxyflavonoids are also one of the main classes of natural flavonoids; they possess antitumor [6], antiviral, and antibiotic effects [7].

Despite lots of recent impressive reports on chalcones [8, 9] and 5-deoxyflavonoids [10], the full potential of such class of compounds is yet to be realized in terms of both more new molecules as drugs and varied biological activity. This situation is largely due to their simple chemical structure and useful template. It has recently become more apparent that most of the important classes of drugs, especially those derived from natural products, are glycosides having a sugar moiety linked to an aglycon through an *O*- or *C*-glycosidic bond. In our continued efforts to use natural products only as synthetic templates and thereby replace the original plant sources with synthetic ones and investigate structure-activity relationship, herein, we wish to report the synthesis and cytotoxicity bioassays of a series of chalcones **1**~**8** and 5-deoxyflabonoids **9**~**16** as well as their glycoside derivatives **17**~**22**. Among them, **13** and **17**~**22** are new compounds.

## 2. Results and Discussion

**Scheme 1:**
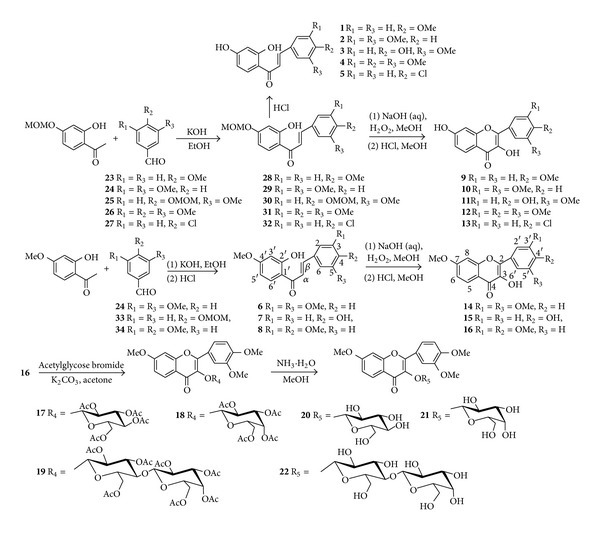


Scheme 1 outlines the synthesis of chalcones **1**~**8 **and 5-deoxyflavonoids **9**~**22**, starting from appropriate benzaldehydes **23**~**27**, **33**, **34**, and acetyl phenols substituted by methoxy or methoxymethoxy, which were purchased or prepared with an improved traditional method in good yields. In the synthesis process, the methoxymethoxy group was chosen to protect the OH group, because it is stable in basic environment and easy to deprotect. Chalcones **28**~**32** were prepared by using aldol condensation of appropriate substituted benzaldehydes **23**~**27**, **33**, **34**, and acetyl phenols in KOH/EtOH and deprotection reaction. The aldol condensation was very sensitive to modification of reaction parameters. A significant excess of KOH (10~15 equiv) was required to force the reaction to completion. Flavonols** 9**~**16** were prepared by classic Algar-Flynn-Oyamada reaction treating the corresponding chalcones with 15% H_2_O_2_ and 16% NaOH (aq) and deprotection reaction.

It is well know that sugar moiety could enhance water solubility and improve the targeting activity of bioactive molecules [11]. For example, lactose can be recognized by the hepatic asialoglycoprotein receptor (ASGP-R), and ASGP-R localized to liver cells provides an efficient entry point for lactose-modified molecules [12]. The modification of 5-deoxyflavonoids with lactose may be possible to specifically target molecules to liver cells, facilitating application of bioactive 5-deoxyflavonoids to the treatment of hepatitis B, hepatitis C, and liver cancer. On the other hand, the largely hydrophobic character of 5-deoxyflavonoids makes it poorly soluble in aqueous media which in some cases limits their therapeutic efficacy, and this has a strong influence on their pharmacokinetic properties. Then, we turned our attention to the introduction of glucosyl, galactosyl, or lactosyl moiety into 5-deoxyflavonoids, and compound **16** was, respectively, condensed with *α*-acetylglucose bromide, *α*-acetylgalactose bromide, or *α*-acetyllactose bromide in the presence of anhydrous K_2_CO_3_ in a solvent of acetone at room temperature to yield the protected 5-deoxyflavonoids glycosides **17~19**. Careful deprotection of the acetyl groups under mildly alkaline condition (NH_3_·H_2_O in MeOH) at room temperature afforded the desired three novel 5-deoxyflavonoids-3-*O*-**β**-D-glycosides **20~22**. The glycosylation selectively affords **β**-products by taking the advantage of 2-OAc neighboring participation effects to secure the 1, 2-trans glycosylation of each sugar residue. In the ^1^H NMR spectra of compounds **20~22**, the chemical shift of the C_1_-H in the glycosyl ring appeared downfield (**δ** 5.5~5.8) with a coupling constant *J*_1,2_ = 7.3 ~ 8.0 Hz, which confirmed their **β**-anomeric configuration [13]. 

The 22 designed target chalcones** 1**~**8** and 5-deoxyflavonoids **9**~**22** were exposed to four human cancer cell lines MDA-MB-23 (human breast cancer cell), U251 (human glia cancer cell), BGC-823 (human stomach cancer cell), and B16 (mouse melanoma cell), respectively, for 48 h using the sulforhodamine B (SRB) protein staining method with the Hydroxycamptothecin (HCPT), Vincristine, and Taxol as positive control. It appeared that these closely related molecules displayed a wide range of inhibitory activities against MDA-MB-23, U251, BGC-823, and B16 cancer cells lines at the maximum concentration of 10 *μ*g/mL as shown in Table 1. Compounds **2**, **4**, **5**, **6**, **10**, **15**, and **19** showed moderate cytotoxic activity against four cancer cell lines with IC_50_ values ranging from 2.37 to 9.71 *μ*g/mL. 

## 3. Experimental

Melting points were measured on a XRC-1 apparatus and were uncorrected. IR spectra were recorded on a Bruker Tensor-27 spectrometer. ^1^HNMR spectra were recorded on a Bruker AM-500 or Bruker AM-400 instrument, using tetramethylsilane as an internal standard, chemical shifts (**δ**) in ppm, and coupling constants (*J*) in Hz. Mass spectra were determined with ZAB-HS spectrometer by the EI or FAB method. Elemental analyses were carried out on a PerkinElmer 240B microanalyser. All solvents were dried by standard procedures. **α**-Acetylglucose bromide, **α**-acetylgalactose bromide, and **α**-acetyllactose bromide were prepared as described in detail [14, 15].

### 3.1. General Procedure for the Synthesis of Chalcones ****1~8****

To a stirred solution of KOH (9.3 g, 165 mmol) in EtOH (40 mL) cooled in an ice bath was added dropwise a solution of the corresponding acetophenone (12.9 mmol) and aldehyde (12.9 mmol) in EtOH (40 mL). The mixture was kept at 0°C for 0.5 h and then room temperature for 22 h. The mixture was poured into ice water (20 mL), adjusted to pH 3~4 with 1 mol·L^−1^ HCl, filtered, and then recrystallized from EtOH to obtain the desired products** 1~8**, respectively.

2′,4′-Hydroxy-4-methoxy chalcone (**1**): light-yellow needles, yield 91%, and m.p. 178~180°C (lit. [16]: 168~170°C). ^1^H NMR (400 MHz, DMSO-*d*_6_): **δ** 3.83 (3H, s, OCH_3_), 6.29 (1H, d, *J* = 2.4 Hz, H-3′), 6.42 (1H, dd, *J* = 9.2, 2.4 Hz, H-5′), 7.03(2H, d, *J* = 8.8 Hz, H-3,5), 7.76-7.77 (2H, d,* J* = 16.0 Hz, H-**α*, *β**), 7.78 (2H, d,* J *= 8.6 Hz), 8.20 (1H, d,* J* = 9.2 Hz, H-6′), 10.71 (1H, s, 4′-OH), and 13.56 (1H, s, 2′-OH); Anal. Calcd for C_16_H_14_O_4_: C, 71.10; H, 5.22. Found: C, 71.32; H, 5.17.

2′,4′-Dihydroxy-3,5-dimethoxychalcone (**2**): yellow needles, yield 92%, and m.p. 96~97°C (lit. [17]: 97~98°C); IR (KBr) *ν*/cm^−1^: 3235, 2967, 1629, 1552, 1281, 1217, 1141, 1059, and 969; ^1^H NMR (500 MHz, acetone-*d*_6_): **δ** 3.83 (6H, s, 2OCH_3_), 6.37 (1H, d, *J *= 2.3 Hz, H-3′), 6.46 (1H, dd, *J *= 8.8, 2.3 Hz, H-5′), 6.57 (1H, s, H-4), 7.01 (2H, s, H-6), 7.78 (1H, d, *J *= 15.4 Hz, H-**β**), 7.92 (1H, d, *J *= 15.4 Hz, H-**α**), 8.12 (1H, d, *J *= 8.8 Hz, H-6′), 9.50 (1H, s, 2′-OH), and 13.20 (1H, s, 4′-OH); MS (FAB^+^) *m/z*: 301 [M+H]^+^.

2′,4′,4-Trihydroxy-3-methoxychalcone (**3**): yellow needles, yield 82%, and m.p. 192~194°C (lit. [16]: 192~194°C); ^1^H NMR (400 MHz, DMSO-*d*_6_): **δ** 3.88 (3H, s, OCH_3_), 6.28 (1H, d, *J* = 2.4 Hz, H-3′), 6.42 (1H, dd, *J* = 8.4, 2.4 Hz, H-5′), 6.83 (1H, d, *J* = 8.4 Hz, H-6′), 7.28 (1H, dd, *J* = 8.8, 1.6 Hz, H-6), 7.53 (1H, d, *J* = 1.6 Hz, H-5), 7.73 (1H, d, *J* = 15.2 Hz, H-**β**), 7.79 (1H, d, *J* = 15.2 Hz, H-**α**), 8.20 (1H, d, *J* = 8.8 Hz, H-2), and 9.74~13.65 (3H, s, OH-2′, 4′, 4); MS (FAB^+^) *m/z*: 287 [M+H]^+^.

2′,4′-Dihydroxy-3,4,5-trimethoxychalcone (**4**): light-yellow needles, yield 94%, and m.p. 188~190°C (lit. [18]: 199~200°C); IR (KBr) *ν*/cm^−1^: 3320, 2912, 2887, 2821, 1619, 1575, 1492, 1455, 1365, 1320, and 1182; ^1^H NMR (400 MHz, DMSO-*d*_6_): **δ** 3.91 (3H, s, OCH_3_), 3.94 (6H, s, 2OCH_3_), 5.79 (1H, s, 4′-OH), 6.44~6.46 (2H, m, H-3′,5′), 6.87 (2H, s, H-2,6), 7.45 (1H, d, *J* = 15.2 Hz, H-**α**), 7.82 (1H, d, *J* = 15.2 Hz, H-**β**), and 13.38 (1H, s, 2′-OH); MS (FAB^+^) *m/z*: 331 [M+H]^+^.

2′,4′-Dihydroxy-4-chlorochalcone (**5**): yellow needles, yield 93%, m.p. 154~156°C (lit. [16]: 156~158°C); ^1^H NMR (400 MHz, CDCl_3_): **δ** 6.33 (1H, d, *J* = 2.4 Hz, H-3′), 6.43 (1H, dd, *J* =8.8, 2.0 Hz, H-5′), 7.54 (2H, d, *J* = 8.8 Hz, H-3, 5), 7.78 (1H, d, *J* = 15.2 Hz, H-**β**), 8.01 (1H, d, *J* = 15.2 Hz, H-**α**), 8.21 (1H, d, *J* = 9.2 Hz, H-6′), and 10.79 (1H, s, 4′-OH); Anal. Calcd for C_15_H_11_ClO_3_: C, 65.58; H, 4.04. Found: C, 65.45; H, 4.10. 

2′-Hydroxy-3,5,7′-trimethoxychalcone (**6**): yellow needles, yield 83%, and m.p. 147~149°C; IR (KBr) *ν*/cm^−1^: 3460, 3009, 2940, 2306, 1639, 1602, 1456, 1370, 1158, and 839; ^1^H NMR (400 MHz, CDCl_3_): **δ** 3.85 (6H, s/each, 3,5-OCH_3_), 3.87 (3H, s, 4′-OCH_3_), 6.48 (1H, d, *J* = 2.8 Hz, H-3′), 6.50 (1H, dd, *J* = 8.4, 2.8 Hz, H-5′), 6.54 (1H, t, *J* = 2.4 Hz, H-4), 6.79 (2H, d, *J* = 2.4 Hz, H-2, 6), 7.53 (1H, d, *J* = 15.6 Hz, H-**β**), 7.80 (1H, d, *J* = 15.6 Hz, H-**α**), and 7.83 (1H, d, *J* = 8.4 Hz, H-6′); MS (EI) *m/z*: 314(M^+^, 100), 297 (10), 283 (13), 177 (60), 164(18), and 151(46).

2′,4-Dihydroxy-4′-methoxy chalcone (**7**): yellow needles, yield 86%, and m.p. 152~153°C; ^1^H NMR (400 MHz, DMSO-*d*_6_): **δ** 3.95 (3H, s, OCH_3_-4′), 6.28 (1H, d, *J* = 2.0 Hz, H-3′), 6.41 (1H, dd, *J* = 9.2, 2.4 Hz, H-5′), 6.84 (2H, d, *J* = 8.6 Hz, H-3, 5), 7.75~7.77 (4H, m, H-**α**, H-**β**, H-2, 6), 8.17 (1H, d, *J* = 9.2 Hz, H-6′), 10.15 (1H, s, 4′-OH), 10.70 (1H, s, 4-OH), and 13.61 (1H, s, 2′-OH); MS (FAB^+^) *m/z*: 271 [M+H]^+^.

2′-Hydroxy-3,4,4′-trimethoxy chalcone (**8**): yellow needles, yield 74%, and m.p. 145~147°C; ^1^H NMR (400 MHz, CDCl_3_): **δ** 3.95 (9H, s, 3OCH_3_), 6.48(1H, s, H-3′), 6.51(1H, d, *J* = 2.8 Hz, H-5′), 6.91 (1H, d, *J* = 8.4 Hz, H-5), 7.16 (1H, d, *J* = 1.6 Hz, H-6), 7.25 (1H, d, *J* = 1.6 Hz, H-2), 7.44 (1H, d, *J* = 15.2 Hz, H-**α**), 7.84 (1H, s, H-6′), 7.86 (1H, d, *J* = 16.8 Hz, H-**β**), and 13.54 (1H, s, OH); MS (FAB^+^) *m/z*: 315 [M+H]^+^.

### 3.2. General Procedure for the Synthesis of 5-Deoxyflavonoids ****9~16****

To a solution of chalcones **6~8**,** 28~32** (0.3 mmol) in methanol (5.5 mL) was, respectively, added 16% NaOH (aq) (0.6 mL), 15% H_2_O_2_ (0.3 mL). The mixture was stirred at room temperature for 24 h, adjusted to pH 3~4 with 1 mol*·*L^−1^ HCl, filtered and then recrystallized from ethanol to obtain the corresponding 5-deoxyflavonols **9~16**, respectively. 

3,7-Dihydroxy-4′-methoxyflavonol (**9**): yellow solid, yield: 68%, and m.p. 289~290°C; ^1^H NMR (400 MHz, DMSO-*d*_6_): **δ** 3.83 (3H, s, OCH_3_-4′), 6.92 (1H, dd, *J* = 2.1, 9.1 Hz, H-6), 6.97 (1H, d, *J* = 2.0 Hz, H-8), 7.03 (2H, d, *J* = 8.8 Hz, H-3′,5′), 7.62 (2H, d, *J* = 8.5 Hz, H-2′,6′), 9.15 (1H, s, OH-7), and 10.74 (1H, s, OH-3); MS (FAB^+^)* m/z*: 285 [M+H]^+^. 

3,7-Dihydroxy-3′,5′-dimethoxyflavonol (**10**): white solid, yield 93.2%, and m.p. 100~101°C; IR (KBr) *ν*/cm^−1^: 3333, 3123, 1741, 1617, 1562, 1274, 1204, 1156, 1064, and 849; ^1^H NMR (500 MHz, DMSO-*d*_6_): **δ** 3.80 (6H, s, 2OCH_3_), 6.63 (1H, s, H-8), 6.91 (1H, d, *J* = 8.8 Hz, H-6), 6.97 (1H, s, H-4′), 7.33 (2H, d, *J* = 1.3 Hz, H-2′,6′), 7.93 (1H, d, *J* = 8.8 Hz, H-5), 9.39 (1H, s, 3-OH), and 10.8 (1H, s, 7-OH); MS (FAB^+^) *m/z*: 315 [M+H]^+^.

7,4′-Dihydroxy-3′-methoxyflavonol (**11**): yellow solid, yield 93%, and m.p. 274~275°C; ^1^H NMR (400 MHz, DMSO-*d*_6_): **δ** 3.85 (3H, s, OCH_3_-3′), 6.91 (1H, dd, *J* = 9.2, 2.0 Hz, H-6), 6.94 (1H, d, *J* = 8.4 Hz, H-5′), 6.98 (1H, d, *J* = 2.0 Hz, H-8), 7.70 (1H, dd, *J* = 8.4, 2.0 Hz, H-6′), 7.77 (1H, d, *J* = 2.0 Hz, H-2′), 7.93 (1H, d, *J* = 9.2 Hz, H-5), 9.13, 9.67 (2H, s/each, OH-4′, 7), and 10.74 (1H, s, OH-3); MS (FAB^+^) *m/z*: 317[M+H]^+^.

3,7-Dihydroxy-3′,4′,5′-trimethoxyflavonol (**12**): yellow solid, yield 85%, and m.p. 120~122°C; ^1^H NMR (400 MHz, DMSO-*d*_6_): **δ** 3.74 (3H, s, OCH_3_-4′), 3.86 (6H, s, OCH_3_-5′, 6′), 6.92 (1H, d, *J* = 6.8, 2.0 Hz, H-8), 7.02 (1H, dd, *J* =8.8, 2.0 Hz, H-6), 7.50 (2H, s, H-2′,6′), 7.93 (1H, d, *J* = 8.8 Hz, H-5), 9.36 (1H, s, OH-7), and 10.78 (1H, s, OH-3); MS (FAB^+^) *m/z*: 345 [M+H]^+^. 

7-Hydroxy-4′-chloroflavonol (**13**): yellow solid, yield 58%, and m.p. 145~146°C; ^1^H NMR (400 MHz, CDCl_3_): **δ** 6.50 (1H, s, OH-3), 6.52~6.54 (2H, d, *J* = 8.4 Hz, H-3,5), 7.09~7.13 (2H, m, H-6,8), 7.60 (2H, d, *J* = 8.4 Hz, H-2,6), and 8.20 (1H, d, *J* = 8.8 Hz, H-5); Anal. Calcd for C_15_H_9_ClO_4_: C, 62.41; H, 3.14. Found: C, 62.22; H, 3.19. 

3-Hydroxy-7,3′,5-trimethoxyflavonol (**14**): yellow needles, yield 49%, and m.p. 197~198°C; IR (KBr) *ν*/cm^−1^: 3449, 3009, 1603, 1556, 1410, 1381, 1263, 1215, 1154, 1120, and 829; ^1^H NMR (400 MHz, CDCl_3_): **δ** 3.89 (6H, s, 3′,5′-OCH_3_), 3.95 (3H, s, 7-OCH_3_), 6.58 (1H, s, H-8), 6.96~7.01 (3H, m, H-6,4′,OH), 7.42 (2H, s, H-2′,6′), and 8.14 (1H, d, *J* = 7.6 Hz, H-5); MS (EI) *m/z*: 328 (M^+^, 100), 313 (10), 297 (19), 285 (30), 178 (10), 149 (23), 122 (16), and 107 (26).

3,4′-Dihydroxy-7-methoxyflavonol (**15**): yellow solid, yield 93%, and m.p. 295~297°C (lit. [19]: 270~272°C); ^1^H NMR (400 MHz, DMSO-*d*_6_): **δ** 3.91 (3H, s, 7-OCH_3_), 6.92~6.96 (2H, m, H-3′,5′), 7.03 (1H, dd, *J* = 8.8, 2.4 Hz, H-6), 7.26 (1H, d, *J* = 2.0 Hz, H-8), 7.98 (1H, d, *J* = 8.8 Hz, H-5), 8.10 (2H, d, *J* = 8.8 Hz, H-2′,6′), 9.23 (1H, s, 3-OH), and 10.07 (1H, s, 4-OH); MS (FAB^+^) *m/z*: 285 [M+H]^+^.

3′,4′,7-Trimethoxyflavonol (**16**): yellow solid, yield 86%, and m.p. 178~180°C (lit. [20]: 185°C); ^1^H NMR (400 MHz, CDCl_3_): **δ** 4.03~3.98 (9H, s/each, 3 OCH_3_), 6.99 (1H, d, *J* = 2.2 Hz, H-6), 7.02 (1H, d, *J* = 2.3 Hz, H-8), 7.06 (1H, s, OH), 7.04 (1H, d, *J* = 3.3 Hz, H-5′),8.17 (1H, d, *J* = 8.9 Hz, H-5), 7.89 (1H, dd, *J* = 8.5, 2.0 Hz, H-6′), and 7.86 (1H, d, *J* = 1.9 Hz, H-2′); MS (FAB^+^) *m/z*: 329 [M+H]^+^. 

### 3.3. Synthesis of 3′,4′,7-Trimethoxyflavonoid-3-*O*-**β**-D-Acetylglucoside ****(17)****

Anhydrous K_2_CO_3_ (150 mg, 1.09 mmol) was added to the mixture of compound **16** (70 mg, 0.2 mmol) and dry acetone (20 mL); then,**α**-acetylglucose bromide (200 mg, 0.49 mmol) was added to the mixture with stirring. After stirring for 12 h at room temperature, the acetone was removed under reduced pressure. The residual was chromatographed on silica gel with petroleum ether/ethyl acetate (3 : 1, volume ratio) as eluent to afford a yellow solid, yield 86%, and m.p. 145~146°C; ^1^H NMR (400 MHz, CDCl_3_): **δ** 2.12~1.89 (12H, s/each, COCH_3_), 3.66~3.61 (1H, m, H-6′′), 3.93 (3H, s, OCH_3_), 3.97 (3H, s, OCH_3_),4.03 (3H, s, OCH_3_), 5.08 (1H, t, *J* = 9.6 Hz, H-4′′), 5.23~5.17 (1H, m, H-5′′), 5.28 (1H, d, *J* = 9.4 Hz, H-3′′), 5.74 (1H, d, *J *= 7.9 Hz, H-1′′), 6.92 (1H, d, *J* = 2.2 Hz, H-2′′), 7.01~6.96 (2H, m, H-6,8), 7.41~7.29 (1H, m, H-5′), 7.67 (1H, dd, *J* = 8.6, 2.0 Hz, H-6′), 7.73 (1H, d, *J* = 2.0 Hz, H-2′), and 8.12 (1H, d, *J* = 8.9 Hz, H-5); Anal. Calcd for C_32_H_34_O_15:_ C, 58.36; H, 5.20. Found: C, 58.61; H, 5.13. 

### 3.4. Synthesis of 3′,4′,7-Trimethoxyflavonoid-3-*O*-**β**-D-Acetylgalactoside ****(18)****

Compound **18** was prepared from **16** and **α**-acetylgalactose bromide as described for the preparation of compound **17** from compound **16** and **α**-acetylglucose bromide. Yellow solid, yield 74%, and m.p. 136~138°C; ^1^H NMR (400 MHz, CDCl_3_): **δ** 1.91~2.16 (12H, s/each, COCH_3_), 3.90~3.98 (9H, 3s, OCH_3_), 4.07 (3H, s, H-2, 4′′, 6′′), 5.15 (1H, dd, *J* = 10.5, 3.5 Hz, H-5′′), 5.39~5.45 (2H, m, H-2′′, 3′′), 5.74 (1H, d, *J* = 8.0 Hz, H-1′′), 6.91 (1H, d, *J* = 2.3 Hz, H-8), 6.97~7.02 (2H, m, H-5′, 6), 7.68 (1H, dd, *J* = 8.6, 2.1 Hz, H-6′), 7.96 (1H, d, *J* = 2.1 Hz, H-2′), and 8.07~8.13 (1H, m, H-5); MS (FAB^+^) *m/z*: 659 [M+H]^+^.

### 3.5. Synthesis of 3′,4′7-Trimethoxyflavonoid-3-*O*-**β**-D-Acetyllactoside ****(19)****

Compound **19 **was prepared from **16** and **α**-acetyllactose bromide as described for the preparation of compound **17** from compound **16 **and **α**-acetylglucose bromide. Yellow solid, yield: 42%, and m.p. 151~152°C. IR (KBr) *ν*/cm^−1^: 3548, 3414, 3139, 1749, 1637, 1618, 1514, 1400, 1237, 1135, 1063, 956, 838, 780, 620, 541, and 484. ^1^H NMR (400 MHz, CDCl_3_): **δ** 1.85~2.15 (21H, s/each, COCH_3_), 3.53~3.55 (1H, m, H-sugar), 3.77~3.92 (3H, m, H-sugar), 3.93~4.01 (9H, s/each, 3OCH_3_), 4.05 (1H, d, *J* = 7.5 Hz, H-sugar), 4.08 (1H, d, *J* = 7.5 Hz, H-sugar), 4.14 (1H, dd, *J* = 6.2, 11.1 Hz, H-sugar), 4.28 (1H, dd, *J* = 12.0, 2.0 Hz, H-sugar), 4.43 (1H, d, *J* = 7.9 Hz, H-1′′′), 4.92 (1H, dd, *J* = 10.4, 3.4 Hz, H-sugar), 5.05~5.13 (2H, m, H-sugar), 5.25 (1H, t, *J* = 9.3 Hz, H-sugar), 5.33 (1H, d, *J* = 2.5 Hz, H-sugar), 5.65 (1H, d, *J* = 7.9 Hz, H-1′′), 6.91 (1H, d, *J* = 2.3 Hz, H-6), 6.95~7.01 (2H, m, H-5′, 8), 7.62~7.68 (2H, m, H-2′, 6′), and 8.11 (1H, d, *J* = 8.9 Hz, H-5); MS (FAB^+^) *m/z*: 947 [M+H]^+^.

### 3.6. Synthesis of 3′,4′,7-Trimethoxyflavonoid-3-*O*-**β**-D-Glucoside ****(20)****

Compound **17** (15 mg, 22.7 mmol) was added to a solution of 30% NH_3_·H_2_O (0.5 mL) in CH_3_OH (3 mL) with stirring. After stirring for 6 h at room temperature, the solvent was removed under reduced pressure. The residual was chromatographed on silica gel with ethyl acetate/EtOH (1 : 1, volume ratio) as eluent to afford a light-yellow solid 70 mg, yield 79%, and m.p. 110~111°C. ^1^H NMR (400 MHz, DMSO-*d*_6_): **δ** 3.11~3.28 (4H, m, H-3′′, 4′′, 5′′, 6′′), 3.37~3.43 (1H, m, H-2′′), 3.57 (1H, dd, *J* = 11.4, 5.6 Hz, H-6′′), 3.85~3.92 (9H, s/each, OCH_3_), 4.38 (1H, t, *J* = 5.4 Hz, OH-6′′), 4.96 (1H, d, *J* = 4.1 Hz, OH-4′′), 5.09 (1H, d, *J* = 4.6 Hz, OH-3′′), 5.43 (1H, d, *J* = 4.2 Hz, OH-2′′), 5.65(1H, d, *J* = 7.3 Hz, H-1′′), 7.08 (1H, dd, *J* = 8.9, 2.3 Hz, H-6), 7.12 (1H, d, *J* = 8.7 Hz, H-8), 7.27 (1H, d, *J* = 2.3 Hz, H-5′), 7.68 (1H, dd, *J* =8.5, 1.9 Hz, H-6′), 7.96 (1H, d, *J* = 1.9 Hz, H-2′), and 7.98 (1H, d, *J* = 8.9 Hz, H-5); Anal. Calcd for C_24_H_26_O_11_: C, 58.77; H, 5.34. Found: C, 58.96; H, 5.28.

### 3.7. Synthesis of 3′,4′,7-Trimethoxyflavonoid-3-*O*-**β**-D-Galactoside ****(21)****

Compound **21** was prepared from compound **18** as described for the preparation of compound **20** from compound **17.** Light-yellow solid, yield 67%, and m.p. 135~137°C. IR (KBr) *ν*/cm^−1^: 3413, 3233, 1618, 1518, 1446, 1399, 1270, 1206, 1077, 1017, 883, 776, 620, and 482; ^1^H NMR (400 MHz, DMSO-*d*_6_): **δ** 3.39~3.46 (3H, m, H-2′, 5′′, 6′′), 3.49 (1H, dd, *J* = 9.7, 4.6 Hz, H-3′′), 3.54~3.61 (1H, m, H-4′′), 3.68 (1H, t, *J* = 3.4 Hz, H-2′′), 3.85 (6H, s, OCH_3_), 3.92 (3H, s, OCH_3_), 4.50 (1H, d, *J* = 5.3 Hz, OH-6′′), 4.54 (1H, d, *J* = 3.7 Hz, OH-2′′), 4.91 (1H, d, *J* = 5.6 Hz, OH-4′′), 5.28 (1H, d, *J* = 4.3 Hz, OH-3′′), 5.57 (1H, d, *J* = 7.7 Hz, H-1′′), 7.09 (2H, dd, *J* = 13.7, 5.4 Hz, H-6,8), 7.28 (1H, d, *J* = 2.2 Hz, H-5′), 7.68 (1H, dd, *J* = 8.5, 1.9 Hz, H-6′), 7.98 (1H, d, *J* = 8.9 Hz, H-2′), and 8.04 (1H, d, *J* = 1.8 Hz, H-5); Anal. Calcd for C_24_H_26_O_11_: C, 58.77; H, 5.34. Found: C, 58.54; H, 5.26. 

### 3.8. Synthesis of 3′,4′,7-Trimethoxyflavonoid-3-*O*-**β**-D-Lactoside ****(22)****

Compound **22 **was prepared from compound **19** as described for the preparation of compound **20** from compound **17**. Light-yellow solid, yield 82%, and m.p. >200°C. IR (KBr) *ν*/cm^−1^: 3413, 3231, 1618, 1588, 1553, 1518, 1447, 1399, 1260, 1223, 1153, 1122, 1091, 1040, 1018, 958, 895, 863, 829, 780, 675, 618, 541, and 484; ^1^H NMR (400 MHz, DMSO-*d*_6_): **δ** 3.38 (2H, s, H-sugar), 3.46 (3H, d, *J* = 7.1 Hz, H-sugar), 3.49~3.59 (3H, m, H-sugar), 3.61 (2H, s, H-sugar), 3.85~3.92 (9H, s/each, OCH_3_), 4.23 (1H, d, *J* = 7.2 Hz, H-sugar), 4.43 (2H, t, *J* = 5.6 Hz, OH-sugar), 4.52 (1H, d, *J* = 4.6 Hz, OH-sugar), 4.68 (1H, t, *J* = 5.1 Hz, OH-sugar), 4.78 (1H, d, *J* = 5.3 Hz, OH-sugar), 4.81 (1H, d, *J* = 1.7 Hz, OH-sugar), 5.09 (1H, d, *J* = 4.3 Hz, OH-sugar), 5.56 (1H, d, *J* = 6.3 Hz, H-1′′′), 5.67 (1H, d, *J* = 7.8 Hz, H-1′′), 7.08 (1H, dd, *J* = 2.4, 8.9 Hz, H-6), 7.13 (1H, d, *J* = 8.7 Hz, H-8), 7.29 (1H, d, *J* = 2.3 Hz, H-5′), 7.71 (1H, dd, *J* = 8.6, 2.0 Hz, H-6′), 7.92 (1H, d, *J* = 2.1 Hz, H-2′), and 7.99 (1H, d, *J* = 8.9 Hz, H-5); Anal. Calcd for C_30_H_36_O_16_: C, 55.21; H, 5.56. Found: C, 55.49; H, 5.47. 

### 3.9. In Vitro Cytotoxic Activity Evaluation by SRB Assay

The cytotoxic activity of the chalcones and 5-deoxyflavonoid was evaluated against MDA-MB-231, U251, BGC-823, and B16 tumor cells. MDA-MB-231, U251, BGC-823, and B16 cells were maintained in RPMI-1640 medium supplement with 10% heat inactivated fetal bovine serum (FBS) and incubated at 37°C in a 5% CO_2_ humidified atmosphere. In order to maintain the cells in log phase cellular suspension, aliquots were refed with fresh RPMI-1640 medium two or three times per week. The stock solutions of the tested compounds were freshly resolved in DMSO and consequently diluted in RPMI-1640. At the final dilutions, the obtained concentration of the solvent never exceeded 0.5%.

The cytotoxic activity was measured in vitro using the SRB colorimetric assay. Cells were inoculated in 96-well microtiter plate (10^4^ cells/well) for 24 h before treatment with the compound(s) to allow attachment of the cell to the wall of the plate. Test compounds were dissolved in DMSO and diluted with saline to the appropriate volume. Different concentrations of the compound under test (0.1, 2.5, 5 and 10 *μ*g/mL) were added to the cell monolayer. Triplicates were prepared for each individual dose. Monolayer cells were incubated with the compound(s) for 48 h, at 37°C, and in atmosphere of 5% CO_2_. After 48 h, cells were fixed, washed, and stained for 30 min with 0.4% (w/v) SRB dissolved in 1% acetic acid. Unbound dye was removed by four washes with 1% acetic acid, and attached stain was recovered with Tris-EDTA buffer. Color intensity was measured in an ELISA reader. The relation between survival curve for cancer cell lines after the specified time. The concentration required for 50% inhibition of cell viability (IC_50_) was calculated.

## Figures and Tables

**Table 1:** IC_50_ value (*μ*g/mL) of chalcones and deoxyflavonoids on the cancer cell lines.

Compound	MDA-MB-231	U251	BGC-823	B16
HCPT^a^	1.18			
Vincristin^b^		0.81		
Taxol^c^			0.002	0.109
**2**	4.97	2.37	3.42	>10
**4**	>10	>10	3.87	2.88
**5**	>10	6.53	3.55	2.66
**6**	6.17	3.37	3.99	>10
**10**	4.21	8.33	3.92	>10
**15**	8.68	4.95	3.71	>10
**19**	5.49	9.71	4.33	4.77

^a,b,c^Used for positive control.
